# Friedrich Dessauer—reflections on his political and personal sacrifices: In reply to: Benzaquen et al. “Friedrich Dessauer (1881–1963): The forgotten medical physicist, politician, and philosopher”

**DOI:** 10.1007/s00066-024-02320-9

**Published:** 2024-11-08

**Authors:** Michael Oertel, Hans Theodor Eich, Oliver Micke, the Working group “History” of the German Society of Radiation Oncology

**Affiliations:** 1 Department of Radiation Oncology, University Hospital Muenster, Muenster, Germany; 2 Department of Radiotherapy and Radiation Oncology, Franziskus Hospital Bielefeld, Bielefeld, Germany

We would like to thank Benzaquen and colleagues for their inspiring review of the biography of Friedrich Dessauer as a remarkable German politician, philosopher, and physicist, who contributed significantly to the field of radiation oncology [1].

However, please allow us to add some additional aspects to further enlighten readers regarding his personal and professional biography.

Friedrich Josef Hubert Dessauer was born in Aschaffenburg into a wealthy, strictly catholic family as the 10th child of Elizabeth Vossen and the industrialist Philipp Dessauer on July 19, 1881 [2–4]. His father ran a factory for colored paper in Aschaffenburg, a third-generation family business founded by his grandfather, Friedrich’s great-grandfather Alois Dessauer, and was himself instrumental in founding the white paper factory. Friedrich Dessauer was thus born into and grew up in very rich and economically oriented surroundings [3, 4].

Friedrich combined his multiple talents with self-dedication and a strong philosophical mind, which helped him throughout his life to pursue political and professional activities [4, 5].

Importantly, Dessauer contributed to important concepts and developments in radiation oncology, such as total body irradiation for which he proposed simultaneous treatment from different radiation sources [2]. He also researched the possibilities of deep therapy, i.e., the treatment of more deeply located tumors with radium and X-rays, and with the development of the crossfire method, he provided the basis for modern cancer treatment [6–8]. With his research into the effects of hard rays on living matter, he founded quantum biology (*Quantenbiologie*) [9].

At the political level, Dessauer joined the conservative catholic *Zentrumspartei* (center party), which he considered to be “the least bad party,” in December 1918 [4]. Shortly afterwards, he was elected party chairman and was active at the community level of politics in Frankfurt am Main [3, 10].

With his election to the German national parliament in 1924, Dessauer aimed to bridge societal conflicts between socialism and Catholicism, the bourgeoisie and the working class. He was dedicated to social politics and a committed republican [3, 4, 10]. As a convinced supporter of the Weimar Constitution, he was one of the few who refused to give their consent to Hitler’s Enabling Act until the very end [3].

Being a social and economic consultant of chancellor Heinrich Brüning, Dessauer was imprisoned and dismissed from political offices after the National Socialists came to power in 1933 [4, 10–12]. Overall, he stands as an example of an active scientist who was perfectly aware of the political challenges of his own time and was not afraid to sacrifice his position in favor of his beliefs.

His former assistant, the biophysicist Boris Rajewsky, took over the leadership of the Institute for the Physical Fundamentals of Medicine that Friedrich Dessauer had previously led, and in 1937 he converted it into the Kaiser Wilhelm Institute for Biophysics under the umbrella of the Kaiser Wilhelm Society [6, 10].

During his 3‑year exile in Turkey, Dessauer established state-of-the-art radiation machines (200 kV for deep treatment, with a 400 kV machine being developed by himself), pioneered the first rotational radiation for laryngeal cancer and a combinational approach between radiation and hyperthermia, and was very active in teaching and science [13, 14].

As he claimed himself, Dessauer was forced to accept his appointment as a professor in Freiburg, Switzerland, after the end of his Turkish contract in 1937, mainly due to his heavy radiation injuries and health problems [13, 15]. He underwent more than 100 skin surgeries during his lifetime, a consequence of his unawareness of the dangers of uncontrolled radiation exposure [3, 4].

In between all these challenges, Dessauer devoted himself passionately to philosophy: “Philosophizing about technology was almost a matter of the heart for Dessauer.” So said Karl Schaezler in his obituary for Friedrich Dessauer [16].

Dessauer practiced this constantly and so it is not surprising that the versatile scholar and prolific writer dedicated a large part of his literary work to evoking the correct understanding of technology and its defense and justification [5, 17].

**Fig. 1:**
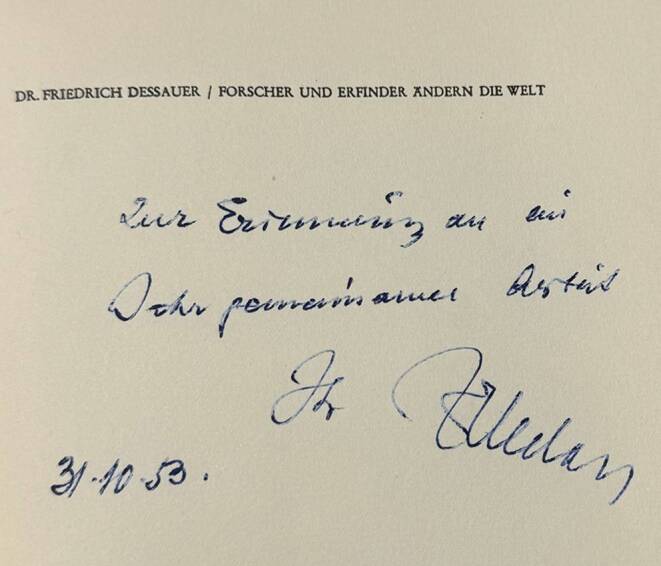
Personal dedication of Friedrich Dessauer in the book *Forscher und Erfinder ändern die Welt* (Collection OM)

The cornerstone of his philosophical thinking was technology as an invention with reflections on the interplay between the inventor and his work ([18–20]; Fig. 1). Dessauer’s focus always remained on the human being, whether as creator or as sufferer [5, 17, 19].

He understood technology in general as a continuation of the creation of God [21]. This does not imply a pantheistic fiction, but rather the reign of the Spirit of God within the world [22]. As an arch catholic Christian, Dessauer was concerned with a religious understanding of technology and the correct integration into his religious worldview [5, 20]. He struggled with the ungodliness of most natural scientists and technicians of his time and strived for a reconciliation of Christianity with modern science and technology [5]. Another repeated motif in Dessauer’s thinking is the service character of technology, thus completing his view [5, 17, 20, 23].

However, during his lifetime, technology entered a new stage of development. New, sensational inventions and especially the trauma left in humanity caused by the atomic bombings triggered a wave of fear and consternation throughout the world in the 1950s—Dessauer himself commented on it in *Nuclear energy and the atomic bomb* (1948) [24]. Just two sentences should be quoted from this document: “Technology has given men a terrible power to destroy, … Woe to the race that received the power but lost the sense” [24].

In 1956, Dessauer’s last major summary of all his efforts in technology was created: *The dispute over technology*, also incorporating his earlier philosophy of technology [25]. He outlined an intellectual historical development and summarized theories and beliefs about the nature, meaning, significance and value [5]. By providing a comprehensive review of positive, negative, and incomprehensible voices in the dispute over technology and illustrating the resulting debate in depth, this book is an example of German scholarship and thoroughness—but for that very reason it is not a really pleasant read [5, 25].

Until then, the knowledge of technical conditions and their intellectual processing was still poor in many circles. But what makes Dessauer’s writings so attractive is his radiant, unbroken idealism and his altruistic attitude towards technology [26]. With Friedrich Dessauer, we continually experience his amazement at the cultural output [5, 25, 27]—a way of looking at things that has unfortunately been lost to those of us living today, for whom technology has become a matter of course; at most, we complain about its imperfections, its risks, and its dangers [28, 29].

Temporal imperfections in technology have been overcome, for which numerous examples are given according to Dessauer [5, 17, 25, 30]. He saw technology as completely inherent and anchored in the human nature. Technology, it is concluded, is not something accidental but rather something deeply essential to humanity [5, 17, 30].

Technology is a means, given and commanded by the sacred Creator, to develop the essence of man [30]. The technician has a real profession in that he follows a call from the Creator’s decree and builds at the foundation of culture [21, 22]. God’s providence reveals itself in technology. On the other hand, Dessauer summarily claims that those who are against technology are so because they want to develop—from some displeasure with technology—a general norm against it, leading from a resentment to a world law, from a misunderstanding to a philosophy, and thereby to a general condemnation of it [5, 17, 25].

Dessauer also always maintained that ethos should be placed above technology [31]. Consequently, he followed the idea that it is unworthy of man to succumb to the spell and fascination of technology, which he deemed a disastrous fatalism based on an unjustified suggestion [5, 17, 25]. Overall, technology is rooted in the nature of reality, and there is an organic technology of life without which human life is not possible. This relaxed view of technology is an enrichment within the theoretic landscape [5, 17, 25, 31].

All in all, Friedrich Dessauer, as a man and as a scientist, adds tremendously to the long triumphant line of physicians, physicists, nurses, and technicians who dedicated their personal health to innovate the field of radiation oncology [32]. Therefore, and for everything he endured, suffered, sacrificed, and lost, both personally and professionally, Friedrich Dessauer is more than worthy of being remembered by all of us.
